# Trauma registry record linkage: methodological approach to benefit from complementary data using the example of the German Pelvic Injury Register and the TraumaRegister DGU®

**DOI:** 10.1186/1471-2288-13-30

**Published:** 2013-03-05

**Authors:** Markus Burkhardt, Ulrike Nienaber, Joerg H Holstein, Ulf Culemann, Bertil Bouillon, Emin Aghayev, Thomas Paffrath, Marc Maegele, Tim Pohlemann, Rolf Lefering

**Affiliations:** 1 Department of Trauma, Hand and Reconstructive Surgery, University of Saarland, Kirrbergerstraße 100, 66421, Homburg/Saar, Germany; 2 AUC - Academy of Trauma Surgery, Landwehrstraße 34, 80336, Munich, Germany; 3 Department of Trauma and Orthopaedic Surgery, University of Witten/Herdecke, Cologne-Merheim Medical Center (CMMC), Ostmerheimerstraße 200, 51109, Cologne, Germany; 4 Institute for Evaluative Research in Orthopedic Surgery, University of Bern, 3014, Bern, Switzerland; 5 Institute for Research in Operative Medicine (IFOM), Faculty of Medicine, University of Witten/Herdecke, 51109, Cologne, Germany

**Keywords:** Trauma registries, Record linkage, Data concordance, Pelvic fracture, Multiple trauma patients

## Abstract

**Background:**

In Germany, hospitals can deliver data from patients with pelvic fractures selectively or twofold to two different trauma registries, i.e. the German Pelvic Injury Register (PIR) and the TraumaRegister DGU® (TR). Both registers are anonymous and differ in composition and content. We describe the methodological approach of linking these registries and reidentifying twofold documented patients. The aim of the approach is to create an intersection set that benefit from complementary data of each registry, respectively. Furthermore, the concordance of data entry of some clinical variables entered in both registries was evaluated.

**Methods:**

PIR (4,323 patients) and TR (34,134 patients) data from 2004-2009 were linked together by using a specific match code including code of the trauma department, dates of admission and discharge, patient’s age, and sex. Data entry concordance was evaluated using haemoglobin and blood pressure levels at emergency department arrival, Injury Severity Score (ISS), and mortality.

**Results:**

Altogether, 420 patients were identified as documented in both data sets. Linkage rates for the intersection set were 15.7% for PIR and 44.4% for TR. Initial fluid management for different *Tile/OTA* types of pelvic ring fractures and the patient’s posttraumatic course, including intensive care unit data, were now available for the PIR population. TR is benefiting from clinical use of the *Tile/OTA* classification and from correlation with the distinct entity “complex pelvic injury.” Data entry verification showed high concordance for the ISS and mortality, whereas initial haemoglobin and blood pressure data showed significant differences, reflecting inconsistency at the data entry level.

**Conclusions:**

Individually, the PIR and the TR reflect a valid source for documenting injured patients, although the data reflect the emphasis of the particular registry. Linking the two registries enabled new insights into care of multiple-trauma patients with pelvic fractures even when linkage rates were poor. Future considerations and development of the registries should be done in close bilateral consultation with the aim of benefiting from complementary data and improving data concordance. It is also conceivable to integrate individual modules, e.g. a pelvic fracture module, into the TR likewise a modular system in the future.

## Background

Pelvic fractures generally have a low incidence of 2-3%, whereas in multiple trauma patients it can rise to more than 25% [1-3]. Because of the high energy that causes a pelvic fracture, the mortality rate for these patients is proportionately high, particularly because of associated head, thoracic, and/or abdominal injuries [3-8]. The recent literature shows a mortality rate of 18% for patients with “complex pelvic injuries”, which include all pelvic fractures (acetabulum, pelvic ring, sacrum) with pelvic soft tissue injuries (i.e., open fractures including the Morel-Lavallée lesion, disruption of pelvic vessels including retroperitoneal hematoma, urogenital or hollow viscus injuries and neurologic deficits directly caused by the pelvic fracture) [1,9,10]. Regardless of the overall encouraging projections of trauma mortality, this rate remains unacceptably high. Scientific explanations for this stagnation are still controversial. In addition to single and multicenter studies, numerous trauma registries worldwide investigate optimal care of the injured patient, not necessarily just those suffering from pelvic fractures. Some such registries are the Victorian Orthopaedic Trauma Outcomes Registry (VOTOR) in Australia, the Trauma Audit and Research Network (TARN) in the United Kingdom, the National Trauma Registry (NTR) of Canada, and the National Trauma Data Bank (NTDB) in the United States. In Germany, we have a special situation in that we have two such registries: the TraumaRegister DGU® (TR) and the unique Pelvic Injury Register (PIR), which focuses on pelvic ring and acetabular fractures. Both registries are surgical driven and are associated with the German Trauma Society (Deutsche Gesellschaft für Unfallchirurgie, or DGU). Because of the differences in focus and content of TR and PIR, many hospitals deliver data to both registries.

**Figure 1:**
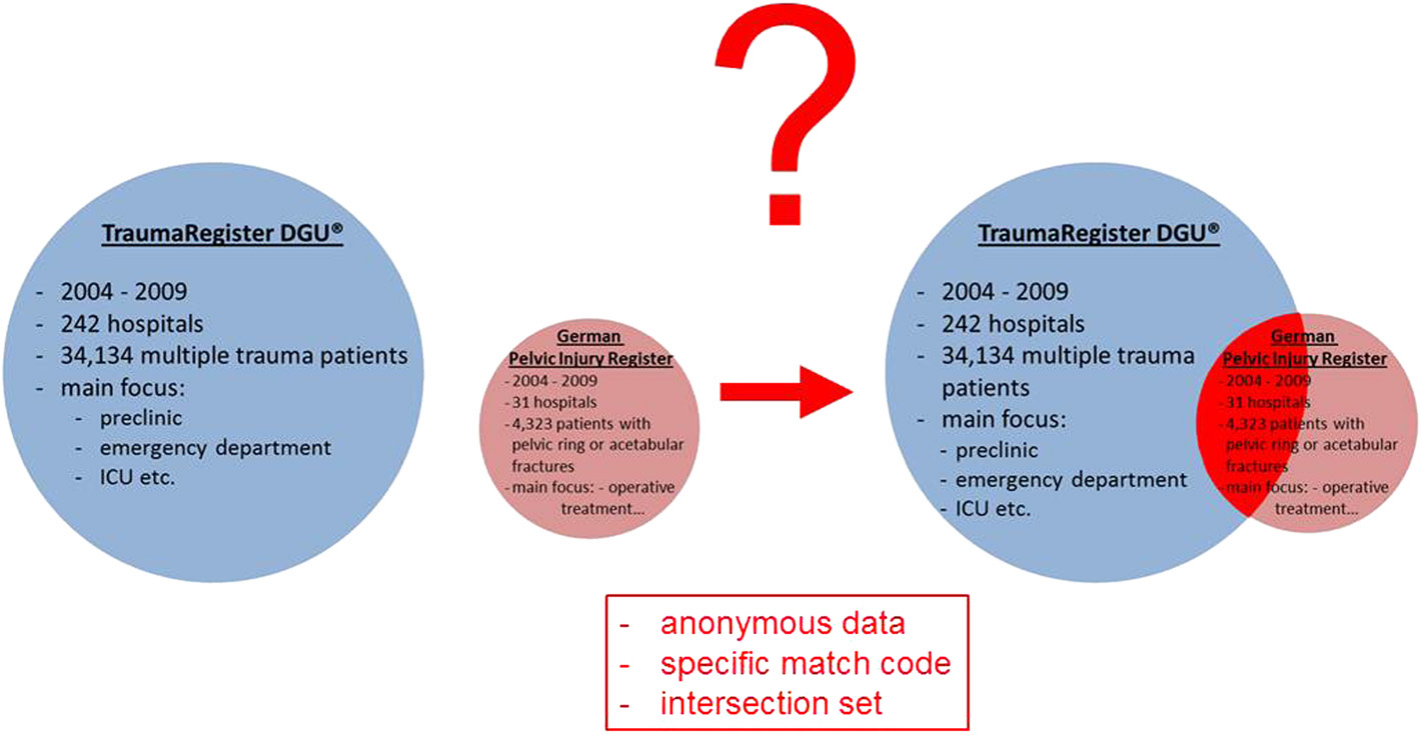
**Documentation of multiple trauma patients in two different trauma registries.** It is possible for multiple trauma patients with a pelvic fracture to be documented in both German trauma registers: Pelvic Injury Register and TraumaRegister DGU®. With the help of a specific match code it was possible to create an intersection set of the two anonymous databases.

We describe the approach to identify identical patients documented in both anonymous trauma registries. We hypothesized that linking the data from the two registries would provide an enlarged data set of information for analysis. Linkage of two anonymous trauma databases to create an intersection set that benefits from complementary data of each registry has not been described previously. Using the PIR and TR as examples, we describe this methodological approach (Figure 1) for the first time. We were especially interested in the extent of concordance of data entries for some of the variables entered in the two registries, including the initial haemoglobin (Hb) and blood pressure (BP) levels on arrival at the emergency department (ED), the Injury Severity Score (ISS), and mortality.

## Methods

### The Pelvic Injury Register (PIR)

In 1991, the PIR started as a joint working group of the German Trauma Society and the German section of the AO Foundation with the prospective, multicentre, standardized and anonymous documentation of patients with pelvic ring or acetabular fractures. It represents the only nationwide database specifically focusing on pelvic trauma and contains detailed information on demographics, fracture classification, in-hospital management with main focus on timing and way of operative treatment, relevant laboratory findings including data on transfusion, and outcome of at least each operative treatment (Table 1). All pelvic fractures were classified by experienced orthopaedic surgeons using the TILE classification adopted by the Orthopaedic Trauma Association (OTA) [11]. Based on plain radiographs and computed tomography scans, stable pelvic ring fractures were classified as a type A injury, fractures with only rotational instability as type B, and fractures with both rotational and translational instability as type C. The PIR is approved by the review board of the German Trauma Society and is in compliance with institutional requirements. In contrast to the TR, the PIR does not keep records of the prehospital phase, and intensive care unit (ICU) data are not abundant. Up to now, at least 36 trauma departments of hospitals from level I-III participated in the PIR during three different observational periods (Working Group I: 1991-1993, Working Group II: 1998-2000, and Working Group III: since 2004). Working Group III started in 2004 with inauguration of a new web-based database that is hosted at the Institute for Evaluative Research in Orthopaedic Surgery, University of Bern (MEMDoc®). In total 31 hospitals are affiliated with Working Group III, which is currently still collecting data. As the PIR is an anonymous registry, the institutional review board waived the need for patient consent.

**Table 1:** **Data contribution to the intersection set of the German Pelvic Injury Register and the TraumaRegister DGU**^**®**^

	**German Pelvic Injury Register (PIR)**	**Intersection set of the PIR and TR**	**TraumaRegister DGU® (TR)**
**Master data**	code of the trauma department, date of the accident, date of admission, date of discharge, birthdate or age, gender, **type of pelvic fracture according Tile/OTA classification, complex pelvic trauma (yes/no)** etc.		code of the trauma department, date of the accident, date of admission, date of discharge, birthdate or age, gender etc.
**Pre-clinic**	none		BP, HR, SaO2, GCS, **intravenous infusions**, rescue times etc.
**Trauma room/Emergency department**	initial hemoglobin, intial BP, PRBC, AIS, ISS, PTS, emergency procedures (i.e. pelvic sling, external fixator, pelvic C-clamp, ORIF etc.) etc.		BP, HR, SaO2, GCS, **intravenous infusions**, diagnostics (x-ray, CT etc.), blood tests, treatment, hemostasis, blood products etc.
**Operation room**	emergency procedures (i.e. pelvic sling, external fixator, pelvic C-clamp, ORIF etc.), **in detail definitive ORIF (pelvic ring or acetabular module, children module)** etc.		emergency procedures (e.g. laparotomy, craniotomy, external fixator etc.), time management etc.
**ICU**	none		SAPS II Score, blood tests, blood products, **ventilation days, complications (sepsis, MOF etc.)** etc.
**Outcome**	date of discharge, complications (e.g. neurological deficits, wound infections, implant failure etc.) etc.		date of discharge, AIS, ISS, OPS-codes for treatment, RISC, TRISS etc.

OTA, Orthopaedic Trauma Association; BP, blood pressure; PRBC, packed red blood cells; AIS, Abbreviated Injury Scale; ISS, Injury Severity Score; PTS, Hannover Polytrauma Score; ORIF, open reduction and internal fixation; HR, heart rate; SaO2, arterial oxygen saturation; GCS, Glasgow Coma Scale; CT, computed tomography; MOF, multiple organ failure; OPS, Operation and Procedure Code; RISC, Revised Injury Severity Classification; TRISS, Trauma Injury Severity Score.

Because of diverging emphases, the registries differ in composition and content. Matching the registries offers the benefit of having complementary data in one database.

### The TraumaRegister DGU® (TR)

The TR was founded in 1993 as a prospective, multicentre, standardized and anonymous documentation of multiple injured trauma patients. Data are collected at four consecutive post-trauma phases from injury to hospital discharge: (i.) pre-hospital phase; (ii.) emergency room/department and initial surgery (until admission to the intensive care unit (ICU)); (iii.) ICU and (iv.) outcome status at discharge and description of injuries and procedures takes place. The registry contains detailed information on demographics, injury pattern, comorbidities, pre- and in-hospital management, time course, relevant laboratory findings including data on transfusion, and outcome of each individual (Table 1). Fluid management is addressed, including records of the infusion volume of crystalloids and colloids started preclinically as well as the blood products transfused during the first 24 hours in hospital. The predominant inclusion criterion is that the injury severity or trauma load requires the patient to stay in the ICU. All injuries are coded using the Abbreviated Injury Scale (AIS). The majority of contributing hospitals are level I trauma centers. In 2009, there were 242 hospitals affiliated with the TR, with an upward trend. The TR is a voluntary register and it is approved by the review board of the German Trauma Society and is in compliance with the institutional requirements. In accordance to the PIR, the TR is a voluntary and anonymous registry that needs no patient consent.

### The linkage procedure of the two data bases

To identify identical patients entered in both registries, a data set was prepared containing the data of all hospitals contributing to both registries during the period 2004-2009. The data set of the TR was then reduced to cases with injuries coded within a specific AIS (1998) numerical injury identifier, i.e. 856xxx.x, containing pelvic ring and acetabular fractures [12]. In the next step, the two data sets were limited to cases where specific hospitals contributed to both registries during the same year. The data sets from the registries were then imported via .csv files into SPSS software (SPSS, Chicago, IL, USA). The unique hospital code used in the TR was introduced into the PIR data set, after which a match-code was created containing the hospital code, dates of admission and discharge, and the age and sex of the patient. Before linking the data sets, each set was checked for possible duplicates. After controlling and (if required) deleting duplicates, the match-code was used to link the two data sets and merged the hits in a new file. This new data file contained the variables of both registries for each patient.

### Checking for data concordance

The following variables were chosen to investigate the concordance of data entries in the two registries: initial haemoglobin and blood pressure levels on arrival at the emergency department, the ISS, and mortality. Records were considered a match if they were identical (the gold standard). Because of the slightly different methods for coding the ISS within the two registries, however, a discrepancy of ± 9 points was tolerated. Also, we accepted discrepancies of ± 1 g/dl for the haemoglobin level and ± 10 mmHg for blood pressure. We considered that there was no substantial impact on clinical treatment or outcome within these ranges.

### Statistical analysis

Data are presented as the mean and standard deviation for continuous variables and as a percentage for categorical variables. The percentage of exact matches and percentage of matches within the tolerated range are given for each variable. Differences in documentation were compared using Wilcoxon test for continuous variables and Chi^2^ for categorical variables. Statistics were calculated using SPSS Statistical Software Package Version 19 (SPSS Inc., Chicago, USA). A p value of <0.05 was considered statistically significant.

## Results

We started with evaluation of the smaller PIR data set, which for 2004-2009 included 4,323 patients’ records collected by at least 31 participating trauma departments. Notably, not all 31 hospitals shared their data with the TR. Ultimately, we found 3,329 anonymous patients from 19 hospitals of the PIR whose data were potentially registered in both databases. In the TR, the initial 34,134 trauma victims from 242 affiliated hospitals during the same observation period were reduced to the same PIR-affiliated hospitals and were screened for the AIS (1998) numerical injury identifier 856xxx.x, reflecting pelvic ring and acetabular fractures.

**Figure 2:**
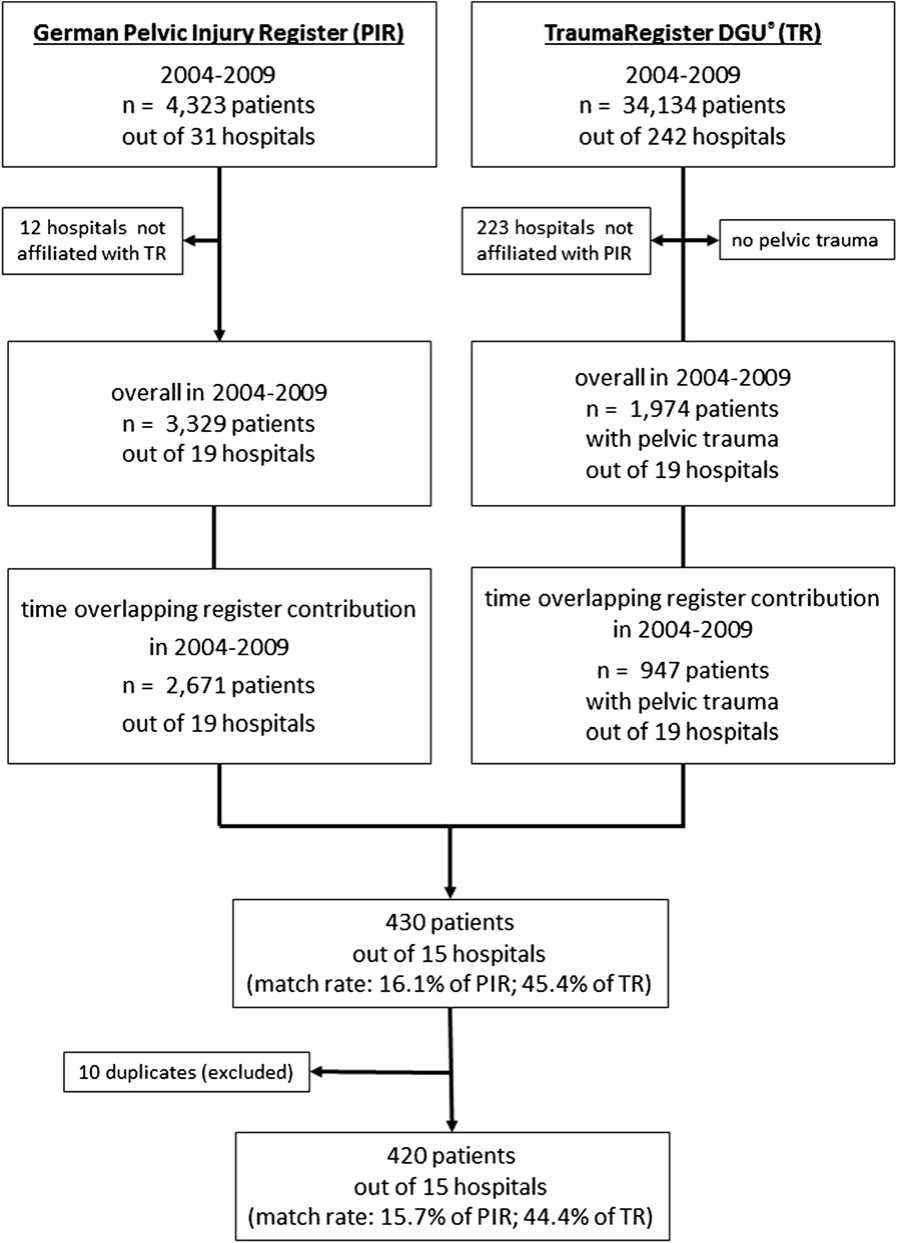
**Process for linking data from the German Pelvic Injury Register and the TraumaRegister DGU^®^.** The linking process identified 420 patients who were documented in both registers during the observation period. The specific match code used for both registers included the codes of the trauma department, date of admission, date of discharge, and the age and sex of the patient.

This search resulted in 1,974 trauma victims with a pelvic fracture. Because of the uneven yearly hospital contributions to the registries, we next focused on the time of overlapping contributions to both registries. It resulted in a further decrease of cases (i.e. 2,671 patients from the PIR, 947 patients from the TR). These patients’ data were linked using a specific match code for both registers including codes for the trauma department, date of admission, date of discharge, and the age and sex of the patient. After exclusion of 10 duplicates, we identified 420 patients with identical match codes (Figure 2).

The data for these 420 patients came from 15 hospitals. Based on the original number of possible patients to identify, we found 15.7% in the PIR and 44.4% in the TR who could be linked. The distribution of the types of pelvic fractures in these linked cases was as follows: 19.8% of type A, 29.5% of type B, 36.4% of type C, and 14.3% of isolated acetabular and/or sacrum fractures. Blunt injury was dominant, with about 99%. The mean age was 41.6 ± 19.5 years, the mean ISS was 27 ± 13 points, and overall mortality was 10%. Complex pelvic injuries were identified in 18.3% (n = 77). For the first time, the initial fluid management for different *Tile/OTA* types of pelvic ring fractures was addressed. Also, ICU and traumatic coagulopathy data were available for the PIR population. On the other hand, the TR is benefiting from the clinical use of the *Tile’s/OTA* classification for pelvic ring fractures and from the correlation with the distinct entity of complex pelvic injuries (Table 1). The individual results of the above-mentioned investigations are already published or will be published separately. The degree of data validation, including data completeness and data concordance, of documented records in both registries is shown in Table 2. Coding of ISS and declaration of patient’s outcome revealed a completion rate of 100% for both registers. In contrast, in the PIR the initial blood pressure and haemoglobin records showed a poor completion rate (17%, 73/420) for both parameters. The completion rates for initial records in the TR were 89% (375/420) for blood pressure and 95% (400/420) for haemoglobin. The low completion rate of 73 of 420 potentially records of initial blood pressure and haemoglobin levels can be explained by the fact that in the PIR these two measurements are mandatory only for complex pelvic injuries - not for benign pelvic ring or acetabular fractures, which do not require emergency fracture management.

**Table 2:** **Degree of data validation of documented records in the German Pelvic Injury Register and the TraumaRegister DGU**^**®**^

	**n [patients]**	**PIR**	**TR**	**Statistics [p]**	**Exact match [%]**	**Tolerance [%]**	**Criteria for tolerance**
**ISS [points, mean ± SD]**	420	27 ± 13	27 ± 13	0.50	13	70	± 9 points
**initial BP [mmHg, mean ± SD]**	73	99 ± 19	108 ± 27	0.005	20	44	± 10 mmHg
**initial Hb [g/dl, mean ± SD]**	73	8.6 ± 2.9	9.6 ± 3.1	<0.001	21	66	± 1 g/dl
**mortality [%]**	420	10% (n = 42)	10% (n = 42)	1.0	100	100	none

PIR, Pelvic Injury Register; TR, TraumaRegister DGU®; ISS, Injury Severity Score; BP, blood pressure on arrival in the emergency department; Hb, haemoglobin on arrival in the emergency department. The exact match of the records was defined as the Gold Standard, but also minor discrepancies according the above-mentioned criteria were tolerated. The low completeness of 73 records of the initial BP and Hb is due to the fact that in the PIR these both parameters are only mandatory for complex pelvic injuries.

## Discussion

### Linkage rate

In multiple trauma patients, the incidence of concomitant pelvic fractures increases up to more than 25% [1-3]. Vice versa, up to 89% of patients sustaining high-energy derived pelvic fractures have at least one associated injury [13,14]. We developed an approach to find patients whose data had been entered into two anonymous trauma registries. Using this approach we identified 420 multiple trauma patients with concomitant pelvic fractures documented in both the PIR and the TR. Prima facie, the linkage rates for the intersection set of 15.7% in the PIR and 44.4% in the TR appear small, which means that only about one in every six patients with a pelvic fracture enrolled in the PIR fulfilled the TR inclusion criterion of “care in the ICU”. On the other hand, approximately every second patient with a pelvic fracture enrolled in the TR was also documented in the PIR. The reason for this discrepancy is that enrolment is mainly determined by the particular emphasis of each registry and the study populations themselves.

Starting with the PIR, the drop-outs might be explained by the large number of isolated acetabular and benign anterior pelvic ring fractures (type A according to the *Tile/OTA* classification) caused by low-energy trauma in the very elderly population [7,10]. Each of these injuries is documented in the PIR but not routinely in the TR because only in rare cases are these patients treated initially as emergency cases in the ICU. Comparing the mean age and the fracture distribution of solely PIR data from Pohlemann et al. with our linked data set, the higher average in age (54.4 years vs. 41.6 years) as well as the higher percentage of type A fractures (42.8% vs. 19.8%) in consequence is found in the PIR [10]. In contrast, the reasons for the even lower drop-outs in the TR are more speculative. Finally, the match code itself could explain the large number of dropouts as there was no tolerance given (e.g. in admission and discharge dates), reflecting an ad hoc deterministic linkage. In contrast to the recently published use of linkage procedures based on encrypted personal identifiers or use of a probabilistic record linkage [15,16], an extensive hand search of both anonymous data sets might have offered the possibility of isolating patients’ data that were mistakenly omitted from one registry and/or to answer the question of how many persons were missed by our linkage criteria. Because of the huge efforts in time and resources, we waived a thorough hand search. This matter, in fact, represents an explicit limitation of our study.

Published articles have compared administrative databases for hospital reimbursement. Trauma registry databases are available for research on epidemiology, practice variation, and outcome [17,18]. There have also been inter-registry analyses in the field of trauma [19,20]. Although the literature is filled with comparisons of these various medical registries, this is the first time that linking data from two clinical trauma registries to create an enlarged data set for each patient has been described. It is also the first time that the benefit of these complementary data has been addressed.

### Data validation

Looking for data validation, we have to address the differences between data completeness and data concordance. Because the ISS and mortality records are directly or indirectly mandatory in both databases, not surprisingly the completion rate was 100% for both parameters in both registries. In contrast, there was a poor completion rate (17%, 73/420) for the initial blood pressure and haemoglobin measurements in the PIR. Usually, this rate is unacceptable compared with the 99% reported in the literature [21]. It should be kept in mind, however, that both parameters are mandatory only for complex pelvic injuries. This appears to be emphasized for the PIR only, reflecting the insistence for vital parameters or the initial haemoglobin level only for complex pelvic injuries. Nevertheless, a high completion rate of the collecting data should be in the interest of each registry, and future data validation schemes must be implemented in the PIR.

The results for data concordance also appear inconsistent. That is, statistics showed no significant differences for the ISS or mortality, but this was not true for the initial blood pressure and haemoglobin measurements. Undoubtedly, the haemoglobin level and hemodynamics of trauma patients influence their outcome and survival. Using only PIR-derived data, Holstein et al. investigated predictors of mortality in patients with pelvic fractures. They found out that the median haemoglobin level of the “survivor group” was 10 g/dl compared with 7 g/dl in the “nonsurvivor group” [8]. Regarding the initial haemoglobin levels of our linkage groups, both mean haemoglobin levels were found to be just between the above-mentioned extremes (8.6 ± 2.9 g/dl from PIR data and 9.6 ± 3.1 g/dl from TR data). Statistics nevertheless showed significant differences, reflecting inconsistency for the data entry level of both clinical parameters. Potential explanations for this inconsistency are numerous (e.g. data entry or coding errors; rounding up or down of numerical values; replicated values during the same treatment period; different error deviations of different haematology analysers or blood pressure-measuring devices).

Finally, by using the defined tolerance levels of these two parameters in the PIR and the TR, we reached the same data entry error rates as in the National Trauma Data Bank (19-76%), although for different records [22-24]. Data quality is still an important challenge for the PIR and the TR. Our results confirmed the need for establishing appropriate validation protocols in the future [24,25].

### Future perspectives and confidentiality concerns

We will also address the use of a unique patient identifier in registers like social security number, which is allowed in some European countries, but not allowed in Germany due to ethical considerations and confidentiality concerns. Such an identifier may considerably help identify a patient across registries, thereby allowing evaluation of the patient’s course through the entire health system. An example is the MEMDoc® module system used in the French hip registry and in other registries that use the social security number even over country borders [26].

## Conclusions

By itself, each trauma registry, i.e. the German Pelvic Injury Register and the TraumaRegister DGU®, reflects a valid source for documenting injured patients in accordance of the emphasis of each registry, respectively. The linkage of these two registries enabled new insights into medical practices for multiple trauma patients with pelvic ring fractures including initial fluid resuscitation and the incidence of traumatic coagulopathy. Inconsistency between clinical PIR and TR data, however, revealed that efforts must be made to ensure high data quality and acceptable population coverage in the future. Present considerations and developments of both registries should take place in close consultation, with the aim of benefiting from complementary data. It is conceivable to integrate individual modules, e.g. a pelvic fracture module, into the TraumaRegister DGU® likewise a modular system. This action would represent a future technical challenge for database programming.

## Competing interests

T. Paffrath and R. Lefering are members of the steering committee of the TraumaRegister DGU®. U. Culemann and T. Pohlemann are members of the steering committee of the German Pelvic Injury Register of the German Trauma Society. Neither of the authors have any other conflicts of interest to declare. Neither this study nor the authors were funded.

## Authors’ contributions

MB, UN and RL had full access to all of the data in the study and take responsibility for the integrity of the data and the accuracy of the data analysis. RL, UN, TPA, and MB conceived and designed the study. JHH, UC, and EA carried out the acquisition of data. UN, RL, and MB performed analysis and interpretation of data. UN, RL, and MB drafted the manuscript. BB, EA, UC, MM, and TPO carried out critical revision of the manuscript for important intellectual content. UN and RL performed statistical analysis. All of the authors read and approved the manuscript.

## Pre-publication history

The pre-publication history for this paper can be accessed here:

http://www.biomedcentral.com/1471-2288/13/30/prepub
